# Between-center variability in the outcome of VLBW infants is not affected by socioeconomic deprivation

**DOI:** 10.1038/s41390-025-03937-x

**Published:** 2025-02-16

**Authors:** Csaba Nador, Andrea Valek, Attila Juhasz, Csilla Nagy, Eszter Bodrogi, Miklos Szabo, Agnes Jermendy

**Affiliations:** 1 Neonatal Intensive Care Unit, Ulloi Street Division, Department of Obstetrics and Gynecology, Semmelweis University, 78/a Ulloi St 1082, Budapest, Hungary; 2 Department of Neonatology, Pediatric Center, MTA Center of Excellence, Semmelweis University, 53-54 Bokay Janos St 1083, Budapest, Hungary; 3 Heim Pál Children’s Hospital, 86 Ulloi St 1089, Budapest, Hungary; 4 National Laboratory for Health Security, Epidemiology and Surveillance Centre, Semmelweis University, 25 Ulloi St 1091, Budapest, Hungary; 5 Neonatal Intensive Care Unit, Central Hospital of Northern Pest – Military Hospital, 109-111 Podmaniczky St 1062, Budapest, Hungary

## Abstract

**Background:**

While outcomes of very low birth weight (VLBW) infants have improved significantly in high-income countries over recent decades, data from Central-Eastern Europe are lacking. The study aimed to evaluate trends in VLBW infant outcomes and hypothesized that a variability exists in the performance of NICUs in Hungary.

**Methods:**

This was a population-based cohort study of VLBW infants, conducted between 2014–2016 (Epoch 1); and between 2019–2021 (Epoch 2) involving all Level III NICUs in Hungary. The primary composite outcome was death or any of the five major morbidities. Adjustments were made for case-mix and hospital-level factors, and the impact of deprivation, a composite index of socioeconomic status, was assessed.

**Results:**

The composite outcome decreased from 39.1% in Epoch 1 (*n* = 3438) to 34.3% in Epoch 2 (*n* = 3084) (*p* < 0.001). Mortality rate reduced significantly by 1.7% (*p* = 0.028). The rate of late-onset sepsis dropped by 4.8% (*p* < 0.001). The adjusted odds of adverse composite outcome decreased 5% yearly (aOR 0.95 (95% C.I. 0.92–0.97)). There was a significant between-center variability in the composite outcome, but it showed no correlation with the deprivation.

**Conclusions:**

Composite outcome trends improved over time, but substantial variability persists among NICUs which cannot be explained by patient characteristics, patient volume, or deprivation.

**Impact statement:**

- Our study presents the first comprehensive, population-based analysis of VLBW infant outcomes in the Central-Eastern European region.
- Composite outcome trends of death and the five major morbidities have shown significant improvement over the past decade among VLBW infants in Hungary.
- A substantial variability exists between the performance of level III NICUs, independent of case-mix and patient volume.
- The odds of adverse outcome in VLBW infants is not correlated with deprivation, a municipal level metric of socioeconomic status.

## Introduction

More than 13.4 million babies are born preterm ( < 37 weeks) every year. Premature birth is a major public health problem worldwide, considered to be the leading risk factor for neonatal mortality.^1^ Postnatal healthcare plays a crucial role in determining the disability adjusted life years (DALY) for premature babies, especially those weighing less than 1500 grams at birth (very low birth weight infants (VLBW)).^2,3^ The greater the immaturity at birth, the higher the vulnerability and incidence of possible complications affecting the central nervous system, lungs, vision, and hearing.

VLBW infants remain in the center of research efforts in neonatal care. The application of evidence-based perinatal (obstetric and neonatal) practices have contributed to decreasing trends of mortality and morbidity in the past 25 years.^4^ Still, even in high-income countries, 33.7–46.9% of this vulnerable population die or survive with one or more major morbidity.^5^ Staying in the Neonatal Intensive Care Unit (NICU) causes significant distress for families.^6^ Additionally, caring for these fragile neonates has an extremely high financial costs imposed on the society.^7^

Large international collaborations such as the Vermont Oxford Network (VON) have helped to benchmark individual NICUs and identify areas for improvement. Analysis of national registries may give more information on regional variation, could aid targeted interventions, and may help to reduce health disparities.

To the best of our knowledge, national data on the outcomes of VLBW infants have not been evaluated in Central-Eastern Europe, although this region comprises 22.3% of the European Union population in 2023.^8^

The premature birth rate in Hungary has remained unchanged over the past decades, standing at 8.3 percent in 2019. This is the second-highest rate in the European Union,^9^ highlighting the urgent need for systematic, nationwide evaluation of care for premature infants.

The aim of our study was to present trends of composite outcome in VLBW infants in Hungary, including mortality and any of the 5 major morbidities: bronchopulmonary dysplasia (BPD), severe intraventricular hemorrhage (IVH grade ≥ 3) or periventricular leukomalacia (PVL), surgical necrotizing enterocolitis (NEC), serious retinopathy of prematurity (ROP), or late onset sepsis (LOS) from two study epochs, 2014–2016 and 2019–2021. We hypothesized that variability exists in the quality of care, as measured by the composite outcome across Level III NICUs nationwide. Our objectives were to explain this variability among centers and over time by examining patient characteristics and hospital-level factors, such as patient volume. Additionally, we investigated whether the ‘deprivation index’, a composite measure of socioeconomic status at the municipality level,^10^ was associated with outcomes of VLBW infants.

## Methods

### Patients

This was a retrospective, population-based, cohort study utilizing data from the Hungarian National In vitro Fertilization, Obstetrical and Perinatal Registry, involving all 21 accredited Level III NICUs in Hungary. The study included neonates born with a gestational age <30 weeks OR birth weight ≤1500 grams. Time trends in overall NICU performance was analyzed in two distinct time epochs: January 2014 to December 2016; and January 2019 to December 2021. The period from 2017 to 2018 had to be excluded from the analysis due to a temporary cessation of data reporting to the national registry. Currently, data reporting is mandated by law. In addition, comparisons between performance of individual NICUs were performed. Exclusion criteria were defined on two levels: NICU level and patient level. Entire NICUs were excluded for (1) low patient volume ( < 10 VLBW neonates/year), (2) non-compliance with mandatory data reporting. Individual patients were excluded for (1) birth weight ≤400 grams or gestational age below 22 weeks, (2) major congenital abnormalities, (3) NICU admission occurred beyond 72 hours of life, or (4) multiple transfers between Level III NICUs up to 72 hours of life. Exclusion criteria (3) and (4) were based on the rationale that infant outcomes and quality-adjusted life years (QALYs) in the VLBW population are predominantly determined by events within the first 72 hours of life. During this critical period, NICU practices play a significant role in shaping these outcomes. However, admissions occurring after this timeframe or multiple transfers between NICUs diminish the ability of any single NICU to exert a substantial influence on the outcome.^11^

### Outcome

The primary outcome was defined as a composite indicator in the Level III NICUs based on the EPIQ study with minor modifications^12^: death or any of 5 major morbidities: BPD (need for supplemental oxygen or respiratory support at 36 weeks corrected gestational age); severe neurological injury (IVH grade ≥3, or IVH grade 2 with hydrocephalus or PVL), NEC requiring surgical intervention (laparotomy or drainage), severe ROP (stage ≥3 or need for treatment in either eye), and LOS (positive results on blood culture or cerebrospinal fluid samples obtained; or clinical sepsis after 3 days of age). As secondary outcomes, we separately evaluated the individual components of the composite indicator. In addition, two sensitivity analyses were performed: one focusing on a subgroup of patients with gestational age less than 30 weeks, and another excluding the NICU that exclusively treats outborn babies.

The outcomes of infants transferred between NICUs (both Level 3 and Level 2, in either direction) were tracked until discharge.

### Deprivation index

The deprivation index is the first composite index in Hungary designed to capture key factors that affect socioeconomic status. The deprivation index is not based on direct, individual data collection, but rather on municipality level factors. The smallest administrative unit in Hungary is the municipality, which refers to a settlement (town or village), rather than a postcode. Currently, there are 3181 municipalities in Hungary, with an average population of approximately 3000 residents. The indicators for the deprivation index are available at the municipality or settlement level. It assesses affluence on a five-point scale, where 1 represents the highest status and 5 the lowest. Deprivation index was calculated of seven municipality level socio-economic indicators from Hungarian Central Statistical Office (Census, 2011) and Hungarian Tax and Financial Control Administration (2011) (income, low qualification, unemployment, one-parent families, large families, density of housing and car ownership).^10^ The weights of indicators obtained from the principal components analysis.

### Statistics

Descriptive statistic of continuous variables is provided as mean with standard deviation. As for binary variables, the rate is described as percentage, with a range (minimum and maximum values) detected on a NICU level. On a patient level, the composite indicator and its components are described as numbers with percentages. The χ^2^ test, Mantel-Haenszel-χ^2^ test for trend and Student’s t test were used to compare clinical characteristics and outcomes between time epochs, as appropriate.

We handled missing data as random and excluded patients from regression analysis if any component of the composite indicator, or any of the confounding variables were missing. The absolute number of missing data was minimal, comprising only 2.5% of the total cohort analyzed.

Univariate and multivariable logistic regression analyses were performed to ascertain the effects of the year of birth on the likelihood of the primary and secondary outcomes, adjusting for clinically relevant confounders, including the following covariates: gestational age in completed weeks and its squared term form, mode of delivery, sex, outborn status, twin status, SGA (birth weight below the 10th percentile for gestational age based on the revised Fenton growth chart for preterm infants,^13^ and 5 minute Apgar score ≤6. These covariates reflect the complexity and severity of VLBW infants, conventionally described as case-mix.

For key outcomes, we followed a three-step approach of analysis and report data in three ways: (1) crude rates, (2) multivariable risk-adjusted models (adjustment based on the individual center’s case mix), and (3) smoothed multivariable risk-adjusted models. This latter modelling technique is also known as random-intercept multilevel (hierarchical) logistic regression modelling.^14^ It allows for separating variability due to patient-level variability (fixed-effects of case-mix) and hospital-level variability (random-effects of patient volume) and applies a shrinkage of the estimates toward the overall mean particularly for the low volume centers. The lower bounds and upper bounds represent the 95% confidence limits around the estimates. The multilevel adjusted odds of composite outcomes were considered statistically significantly different, if the 95% confidence intervals did not overlap.

Finally, we evaluated the association between the deprivation index on a 5-point scale, and the occurrence of composite outcome in Epoch 2 using Mantel-Haenszel χ^2^-test to assess trends. Correlation between the deprivation index on a five-point scale and the odds of composite outcome was assessed with Spearman’s rank correlation coefficient (Spearman’s ρ).

SPSS Statistics version 27 (IBM, Armonk, NY) and Prism version 10.1.0 (GraphPad Software, Boston, MA) were used for data analysis and plotting.

## Results

A total of 6822 neonates born with gestational age <30 weeks or birth weight ≤1500 grams admitted to one of the 21 Level III NICUs in Hungary between 2014–2016 (Epoch 1, *n* = 3657) or 2019–2021 (Epoch 2, *n* = 3165) were eligible for this study. We noted a 13.5% decrease in admission volume in Epoch 2.

**Fig. 1:**
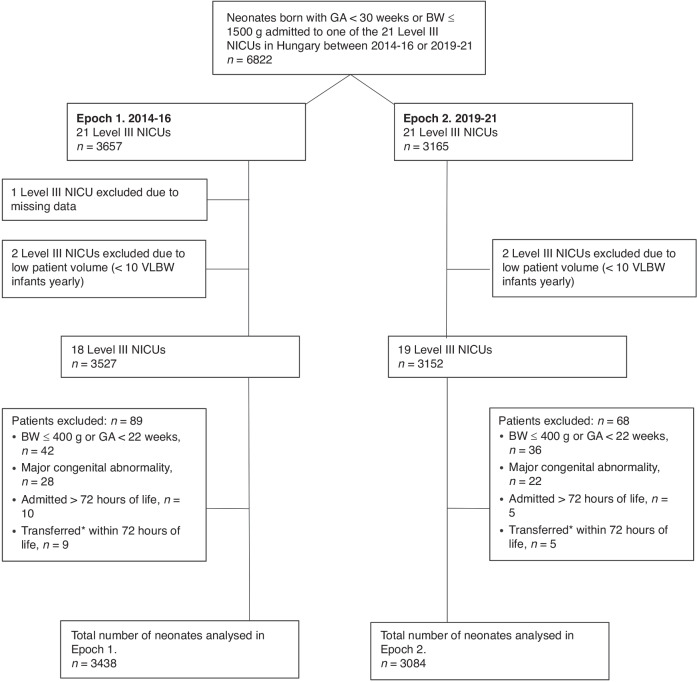
Flow-chart of the study population. ^∗^Neonates transferred multiple times within 72 hours or transferred once and died within 72 hours or transferred to a step-down unit (Level II NICU) within 72 hours were excluded from the analysis.

Of the 21 Level III NICUs, one center was excluded in Epoch 1 due to incomplete reported data. Additionally, two centers were excluded in both Epoch 1 and 2 that cared for less than 10 infants annually during the study period (Fig. 1).

Patients with a birth weight ≤400 grams or gestational age below 22 weeks, major congenital abnormalities, admission beyond 72 hours of life or multiple transfers between level III NICUs within 72 hours of life were excluded. We believe that these exclusions were essential to evaluate individual NICU performances independently of patient characteristics not influenced by the standards of care, such as borderline viability or the burden of neonatal transfers. After exclusions (*n* = 219 in Epoch 1 and *n* = 81 in Epoch 2), a total of 3438 and 3084 infants were analyzed in Epoch 1 and Epoch 2, respectively. Infant characteristics were similar in the two study periods, except for the significantly increased rates of outborn infants and 5-minute Apgar scores ≤ 6 by Epoch 2 (Table 1).

**Table 1:** Demographic profile of the study population in the two time epochs.

	2014–2016 (*n* = 3438)	2019–2021 (*n* = 3084)	*P* value
Admission volume per NICU per year, number of infants, median (range)	47 (15–173)	34 (9–131)	
Gestational age, weeks, mean (SD)	28.8 ± 3.0	28.9 ± 3.0	0.621
Birthweight, grams, mean (SD)	1110 ± 315	1125 ± 310	0.232
Small for gestational age, %, mean (range)	24.4 (13.8–33.1)	24.6 (14.4–31.7)	0.854
Outborn, %, mean (range)	9.7 (0.0–100.0)	11.3 (0.0–100.0)	**0.029**
Caesarean section, %, mean (range)	82.1 (60.9–90.3)	81.5 (66.1–90.6)	0.517
Antenatal corticosteroid, %, mean (range)	75.9 (35.6–87.5)	75.0 (38.8–88.3)	0.997
Male sex, %, mean (range)	50.3 (38.3–57.5)	49.7 (43.2–56.7)	0.620
5 minute Apgar score ≤ 6, %, mean (range)	12.0 (2.1–26.9)	15.4 (5.3–33.9)	**<0.001**
Multiple Birth %, mean (range)	29.6 (12.6–37.1)	28.1 (6.8–37.1)	0.190

Significant p values are set in bold.

The composite outcome of death or any of the 5 major morbidities decreased from a mean rate of 39.1% in Epoch 1 to 34.3% in Epoch 2 (*p* < 0.001) (Table 2). There was a variability among centers regarding the initial crude composite outcome rates and their improvement over time; of the 18 NICUs with complete dataset in both Epochs, 14 improved or had similar crude composite outcome rates in the two study epochs. We also assessed the individual components of the composite outcome (Table 2). The mortality rate decreased significantly from 11.9% in Epoch 1 to 10.2% in Epoch 2 (*p* = 0.028), but not in all centers. Of the 5 major morbidities, the crude rate of BPD increased significantly by 1.7% (*p* = 0.008), and LOS decreased by 4.8% in Epoch 2 (*p* ≤ 0.001).

**Table 2:** Crude rates of the outcome variables in the two study epochs within the entire VLBW study population.

	2014–2016 (*n* = 3438)	2019–2021 (*n* = 3084)	*P* value
Composite outcome rate (range)	39.1 (21.9–66.3)	34.3 (17.3–64.4)	**<0.001**
Mortality rate (range)	11.9 (5.8–21.3)	10.2 (1.0–27.1)	**0.028**
BPD rate (range)	6.3 (1.2–13.8)	8.0 (0.0–18.6)	**0.008**
Severe IVH/PVL rate (range)	13.2 (4.3–26.9)	12.3 (2.9–32.2)	0.254
Surgical NEC rate (range)	2.8 (0.0–6.9)	3.0 (0.0–13.6)	0.543
Severe ROP rate (range)	7.7 (2.1–12.5)	7.4 (0.0–17.3)	0.646
LOS rate (range)	22.1 (5.1–51.3)	17.3 (5.8–45.8)	**<0.001**

Numbers show the rate of occurrance in the total patient population, and the range of rates in the studied NICUs.

Significant changes over time are set in bold.

*BPD* bronchopulmonary dysplasia, *IVH* intraventricular hemorrhage, *PVL* periventricular leukomalatia, *NEC* necrotizing enterocolitis, *ROP* retinopathy of prematurity, *LOS* late onset sepsis.

See text for details.

**Fig. 2:**
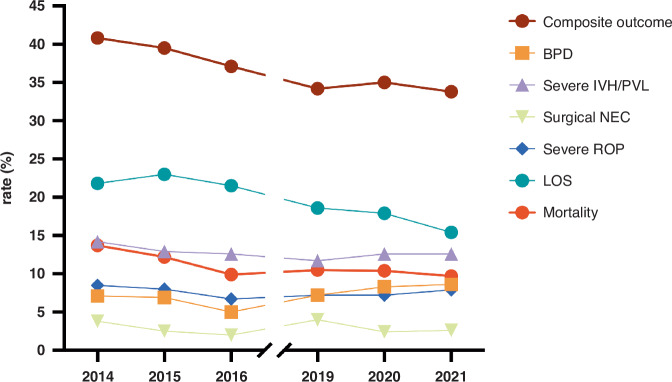
The unadjusted rate of composite outcome and individual morbidities and mortality over the study period in eighteen NICUs. OR odds ratio, aOR adjusted odds ratio, BPD bronchopulmonary dysplasia, IVH intraventricular hemorrhage, PVL periventricular leukomalacia, NEC necrotizing enterocolitis, ROP retinopathy of prematurity, LOS late onset sepsis.

Yearly trends of composite outcome and major morbidities are shown in Fig. 2. Logistic regression analyses revealed that the year of birth had a significant effect on the likelihood of the primary composite outcome, and the secondary outcomes separately (Supplemental Table 1). Specifically, there was a 5% decrease in the odds of adverse composite outcome yearly (adjusted odds ratio (aOR) 0.95 (95% confidence interval (C.I.) 0.92–0.97). Similarly, the odds of mortality and LOS both decreased by 6% yearly (aOR 0.94 (95% 0.91–0.98) and aOR 0.94 (95% C.I. 0.92–0.97), respectively). On the contrary, the odds of BPD increased by 6% yearly (aOR 0.94 (95% C.I. 1.02–1.10). The odds of other morbidities did not change over time.

**Fig. 3:**
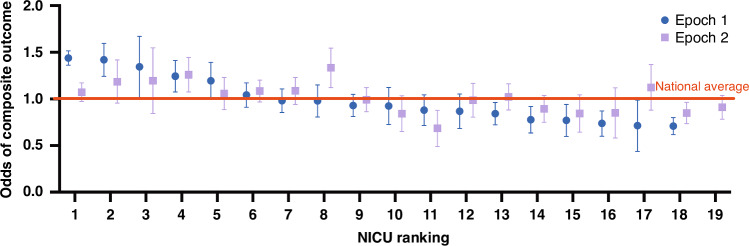
NICUs ranked according to the odds of composite outcome with respect to the national average using multilevel risk-adjusted regression modelling. NICUs are numbered from 1 to 19. Blue circles represent estimates for each NICU in Epoch 1 and purple rectangles represent Epoch 2. Whiskers represent the 95% confidence limits around the estimates.

Next, we sought to compare individual NICU outcomes using regression modelling. As expected, several patient level fixed-effect covariates describing the case-mix were significantly associated with the composite outcome in the risk adjusted models in the total study population in both epochs, including gestational age, small for gestational age status and the 5-minute Apgar score ≤6. Moreover, the multilevel logistic regression model adjusted for case-mix and the volume of admission revealed that there was a significant between-center variability in the composite outcome (Fig. 3). In Epoch 1, four centers (marked as no. 1–4) had significantly increased odds for adverse composite outcome compared to the national average, and one center improved significantly by Epoch 2. There were 6 centers (marked as no. 13–18.) who had achieved better outcomes than the national average in Epoch 1, demonstrating a huge variability in NICU performance across Hungary. In contrast, by Epoch 2, only 4 centers had significantly different performance than the national average (no. 4, 8, 11, 18) suggesting a somewhat decreased variability. Of note, only 2 centers (no. 8 and 17) had tendency for worsening outcomes in Epoch 2, but these changes were not significant.

**Fig. 4:**
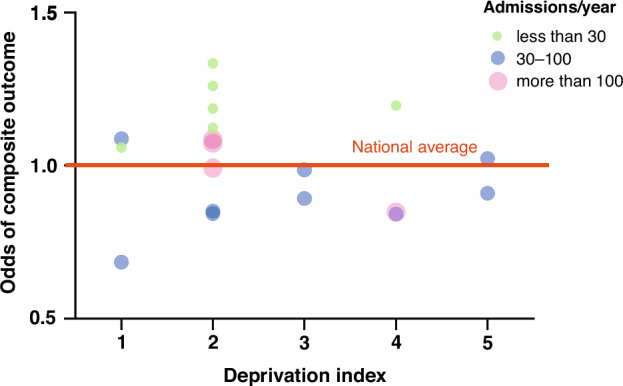
Relationship between the odds of composite outcome in Epoch 2, NICU size and the median deprivation calculated for the individual NICUs based on municipalities across the region of care. Green circles represent small NICUs with less than 30 admissions yearly, blue circles represent medium-size NICUs with 30-100 admissions yearly, and red circles represent large NICUs with more than 100 admissions yearly.

To understand the variability in outcomes and improvement among NICUs we assessed the possible role of socioeconomic status using the deprivation index, as a proxy metric, in Epoch 2, the period when data from all 19 large NICUs were available. On patient level, we found no association between deprivation index, categorized on a five-point scale according to the municipality of residence for each family, and the incidence of the composite outcome (Mantel-Haenszel χ^2^-test for trend, *p* = 0.379). Also, on NICU level, there was no correlation between the odds of composite outcome and the median deprivation index of the region of care (Spearman’s ρ=−0.180, *p* = 0.461). Figure 4 shows the relationship between the odds of composite outcome, admission volume and deprivation categories for each NICU. Even after adjusting for patient volume in the multilevel regression analysis, it is noticeable that NICU performance was found to be unrelated to the deprivation index of the populations they served but was instead associated with NICU size.

Finally, two sensitivity analyses were performed. First, we analyzed patients with gestational age <30 weeks only. We found similar trends to the whole patient population with respect to changes in composite outcome as well as individual morbidities, although absolute crude rates were higher in accordance with more vulnerability of this subgroup (Supplemental Table 2). Composite outcome rates decreased from 55.8% to 51.1% (*p* = 0.003), in parallel with a significant reduction in LOS, and increase in BPD. Other morbidities did not change over time. In Hungary, there is only a single NICU that exclusively treats outborn babies. We conducted a sensitivity analysis excluding this center (Supplemental Table 3) and noted similar outcomes.

## Discussion

The composite outcome rate of death or any of the 5 major morbidities has improved significantly among VLBW infants in Hungarian NICUs between 2014–2016 and 2019–2021. Despite this overall progress, significant variability persists between centers, even after adjusting for patient characteristics and admission volume. While most centers improved over time, some centers had unchanged or reversed outcome metrics. The heterogeneity of center outcomes could not be explained by municipality-level socioeconomic deprivation,^10^ suggesting that other unmeasured NICU characteristics play a critical role in the distinct outcomes. Our findings provide valuable benchmarks for individual centers in Hungary and have the potential to guide future quality improvement efforts.

Our study represents the first comprehensive, population-based analysis of VLBW infant outcomes in the Central-Eastern European region. While a previous smaller report indicated higher morbidity and mortality rates in the Baltic region,^15^ more recent data were lacking. In our study, we used a composite outcome in VLBW infants to describe temporal and between-center changes as this is a widely adopted, pragmatic indicator of NICU performance.^16^ Notably, composite metrics are mostly used by North American study groups.^12,17^ The rationale for using a composite metric is that the quality improvement efforts typically influence multiple outcomes simultaneously, allowing for a more comprehensive assessment of overall changes. Over the 8-year study period, we observed an improvement in patient outcomes, similarly to other reports.^18–20^ The progress was mainly driven by the decrease in LOS over the study period. These favorable trends were likely attributed to the clinical implementation of recent scientific evidence and the dissemination of best perinatal practices. However, no nationwide, structured, or continuous quality improvement projects were conducted during the study period. In other words, our study highlights the incremental improvements in survival and reduction in morbidity among VLBW infants that can be achieved, even in the absence of targeted quality improvement initiatives in the Central-Eastern European region.

Individual morbidity trends are consistent with international findings. One notable difference is that the rate of bronchopulmonary dysplasia appears to be very low (6.3–8.0%). Although the rate of BPD slowly increased over time, most likely due to the improved survival of the smallest infants, we were unable to identify a single factor explaining the surprisingly low incidence rates. We used the definition of receiving supplemental oxygen or any other form of respiratory support at 36 weeks’ postnatal age for BPD, which is widely used in research and clinical reporting. Patient inclusion criteria in our study followed the VON eligibility criteria,^21^ specifically we included infants with ≤1500 grams birth weight or <30 weeks of gestational age. Therefore, it is plausible that SGA infants with higher gestational age and lower birth weight may have contributed to the favorable BPD rates observed. Notably, the incidence of SGA was approximately 25% in both study periods within our cohort. To further explore this, we conducted a sensitivity analysis restricting to infants with <30 weeks of gestation. As anticipated, the crude rates of the outcome variables were higher in this group but demonstrated similar trends across the two study epochs. For BPD, we found an increased rate from 10.0% to 13.0% in the <30 weeks subgroup. This trend aligns with findings from the Israeli Neonatal Network between 2000–2010 (13.7%),^22^ the Canadian (12.3%) and the Japanese Neonatal Networks (14.6%) during 2006–2008.^23^ In contrast, reports from the VON database describe a higher yearly rate of BPD for VLBW infants, ranging from 26.2% to 30.4% between 2000–2009.^4^ Clearly, differences in study periods and patient populations make direct comparisons of outcomes among survivors between our study and those from other countries challenging. However, the trends indicate that BPD remains a major complication among VLBW infants, requiring substantial post-discharge care, even as survival rates continue to improve.

The incidence rate of severe IVH was relatively high in our patient population (13.2–12.3%) and, unfortunately, remained unchanged over time. Our findings emphasize the urgent need for a national quality improvement initiative focused on IVH prevention. Such a project could involve the implementation of care bundles, collaborative learning and a “learning by doing” approach tailored to the local context.^11^ These strategies have been demonstrated to be effective in driving improvements and could significantly enhance quality-adjusted life years for both patients and their families.

The incidence rate of severe necrotizing enterocolitis requiring surgical intervention in our study is consistent with the data reported by Bell et al.^20^ We attribute this favorable complication rate to the widespread nationwide practice of early administration of breast milk/donor milk and the routine use of probiotics.

The incidence of severe retinopathy of prematurity (ROP), which is similar to the international data has not decreased significantly over the examined period.^5^

Late onset sepsis (LOS), including central line-associated bloodstream infection (CLABSI) remains one of the greatest challenges faced by NICUs worldwide. Of note, our definition of LOS included both clinical sepsis and culture-proven infections, which may have broadened the patient population and diluted the impact of culture-proven cases. Currently, there is a lack of consensus on neonatal sepsis definition. However, our approach to defining LOS aligns with a recent systematic review, which highlights that the most widely used components of neonatal sepsis diagnosis worldwide are definite, culture-proven sepsis and probable, clinical sepsis.^24^ During hospitalization, the mortality rate of VLBW infants most frequently increases due to complications associated with LOS.^25,26^ In our study, we detected a significant decrease in the occurrence of LOS between the two examined periods, however, it is worth pointing out that the absolute rates exceed 20 percent, with a wide variation of 5 to 50% among centers. The Swiss Neonatal Network and US members of the Vermont Oxford Network report a LOS incidence rate of less than 10 percent.^5^ The high rate of LOS underscores the need for future quality improvement projects, including the adaptation of care bundles^27,28^ aimed to reduce LOS in this vulnerable patient population. Furthermore, the definition of LOS should be carefully reviewed, and the mandatory implementation of a specific NICU-compatible surveillance system such as the German NEO-KISS could play a critical role in helping VLBW infants survive without nosocomial infections.^29^

A novel aspect of our study is the assessment of whether the significant outcome variability among NICU centers could be associated with the social background of VLBW infants. According to a recent article from California, multilevel social factors significantly influence the quality of care in NICUs. Facilities with a higher proportion of minority racial/ethnic patients and patients from lower socioeconomic status (SES) backgrounds were found to have lower quality scores.^30,31^ Health records in Hungary do not include data on racial background. Therefore, we used the deprivation index to perform the comparison. While this index does not reflect the unique social status of individual families, it captures the welfare indicators of the municipalities. During the COVID-19 pandemic, deprivation index proved to be a reliable metric for assessing health outcomes. Patients living in more deprived municipalities found to have a lower risk of being identified as confirmed COVID-19 cases and a higher risk of death.^32^ Interestingly, in the present study, deprivation was not associated with differences in composite outcomes. Therefore, the variability among centers could be explained by other unmeasured covariates such as staffing models, physical infrastructure, and cultural aspects of the NICUs.^33^

There are several limitations of our study that should be acknowledged. First, the size of individual centers and their patient volumes varied considerably. To address this variability, we applied statistical adjustments by employing shrinkage toward the national mean using multilevel logistic regression. This method is widely accepted for reducing variability caused by the small sample sizes of certain centers.^34^ However, some may argue that it could underestimate the typically poorer outcomes of low-volume, low-quality providers.^35^ In the present study, even after adjustments for patient volume, small NICUs had a tendency for increased odds of adverse outcome. Second, the case-mix of NICUs varied as there is a center that cares for outborn babies exclusively, and only few NICUs have surgical patients. To handle selection bias, statistical adjustments were applied to account for these patient characteristics and reduce variability in the analysis. Third, we were unable to investigate the potential impact of delayed umbilical cord clamping, administration of breast milk and developmental care, as these variables were not routinely collected. Additionally, antenatal steroid administration was not consistently reported, preventing us from including this important covariate in our models. Finally, when adjusting for socioeconomic status of families, we were limited to using the municipality-level deprivation index, rather than individual-level metrics. However, it has been demonstrated that deprivation indices at the municipal level are excellent predictors of health outcomes in the adult population.^10,32^ We believe that as survival rates continue to improve and family-centered care becomes more widespread, social determinants of health will play an increasingly significant role in shaping the outcomes of VLBW infants in the future.

In conclusion, the composite outcome of death and five major morbidities have significantly decreased among VLBW infants in Hungary over the 2014–16 and 2019–21 period. However, marked variability persists among NICUs, which could not be fully explained by patient characteristics, patient volume or the deprivation. Targeted, national quality improvement initiatives are urgently needed to enhance outcomes, especially for IVH and LOS, and to mitigate variability across centers. All VLBW infants deserve the opportunity to achieve the highest possible quality-adjusted life years.

## Supplementary information

Supplementary Material

## Data Availability

The data supporting the findings of this study are not publicly available due to sensitivity concerns. However, they can be obtained from the corresponding author upon reasonable request.
