# Supplementary material for: Between-center variability in the outcome of VLBW infants is not affected by socioeconomic deprivation
Source: Pediatr Res. 2025 Feb 16;98(4):1323–30. doi: 10.1038/s41390-025-03937-x (PMC12549325; doi:10.1038/s41390-025-03937-x)
Supplement: Supplementary file 1 — Supplementary Material [file 41390_2025_3937_MOESM1_ESM.pdf]

Supplemental table 1. Logistic regression analyses assessing the effect of the year of birth on the likelihood of composite adverse outcome, mortality and the 5 major morbidities. Univariate and multivariable adjusted models are shown.

	OR	95% C.I.	P value	aOR	95% C.I.	P value
**Composite outcome**	0.959	0.941-0.978	**<0.001**	0.946	0.924-0.969	**<0.001**
**Mortality**	0.958	0.930-0.986	**0.004**	0.939	0.905-0.975	**0.001**
**BPD**	1.044	1.007-1.082	**0.018**	1.062	1.023-1.104	**0.002**
**Severe IVH/PVL**	0.983	0.956-1.011	0.225	0.989	0.959-1.020	0.469
**Surgical NEC**	0.989	0.936-1.045	0.690	0.999	0.944-1.059	0.985
**Severe ROP**	0.988	0.954-1.023	0.508	1,000	0.963-1.038	0.988
**LOS**	0.942	0.920-0.964	**<0.001**	0.943	0.920-0.967	**<0.001**

Abbreviations: OR, odds ratio; aOR, adjusted odds ratio; BPD, bronchopulmonary dysplasia; IVH, intraventricular hemorrhage; PVL, periventricular leukomalatia; NEC, necrotizing enterocolitis; ROP, retinopathy of prematurity; LOS, late onset sepsis.
Multivariable logistic regressions were adjusted for the following covariates: gestational age, sex, twin status, small for gestational age status, 5 min Apgar score less than 7, outborn status, mode of delivery (vaginal birth vs caesarian section).

Supplemental table 2. Sensitivity analysis, showing the crude rates of the outcome variables in a subgroup of patients with less than 30 weeks of gestation in the two study epochs.

	**Sensitivity analysis (subgroup with <30 weeks of gestation)**		
	**2014-2016** n=2031	**2019-2021** n=1755	**P value**
Composite outcome rate (range)	55.8 (38.1-90.2)	51.1 (34.5-70.0)	**0.003**
Mortality rate (range)	18.8 (8.9-31.4)	16.5 (3.2-37.0)	0.060
BPD rate (range)	10.0 (1.9-19.6)	13.0 (0.0-35.5)	**0.004**
Severe IVH/PVL rate (range)	20.1 (5.3-37.2)	19.3 (6.5-40.0)	0.523
Surgical NEC rate (range)	4.0 (0.0-10.3)	4.8 (0.0-12.5)	0.262
Severe ROP rate (range)	12.7 (3.9-26.3)	12.1 (0.0-23.1)	0.644
LOS rate (range)	29.8 (8.3-66.7)	25.2 (11.7-51.4)	**0.002**

Numbers show the rate of occurrance in the total patient population, and the range of rates in the studied NICUs.
Abbreviations: BPD, bronchopulmonary dysplasia; IVH, intraventricular hemorrhage; PVL, periventricular leukomalatia; NEC, necrotizing enterocolitis; ROP, retinopathy of prematurity; LOS, late onset sepsis; wk, weeks of gestation.
Significant changes over time are set in bold.
See text for details.

Supplemental table 3. Sensitivity analysis, excluding the NICU that cares exclusively for outborn neonates. The crude rates of outcome variables among VLBW patients are reported in the two study epochs.

	**Sensitivity analysis (subgroup excluding the NICU caring for outborn neonates)**		
	**2014-2016** n=3351	**2019-2021** n=3025	**P value**
Composite outcome rate (range)	38.6 (21.9-66.3)	33.8 (17.3-49.3)	**<0.001**
Mortality rate (range)	11.7 (5.8-21.3)	9.9 (1.0-23.3)	**0.020**
BPD rate (range)	6.4 (1.2-13.8)	7.8 (0.0-18.6)	**0.024**
Severe IVH/PVL rate (range)	13.0 (4.3-26.9)	11.9 (2.9-22.0)	0.203
Surgical NEC rate (range)	2.7 (0.0-6.0)	2.8 (0.0-5.1)	0.706
Severe ROP rate (range)	7.8 (2.1-12.5)	7.3 (0.0-17.3)	0.480
LOS rate (range)	21.7 (5.1-51.3)	16.8 (5.8-45.8)	**<0.001**

Numbers show the rate of occurrance, and the range of rates in the studied NICUs.
Abbreviations: BPD, bronchopulmonary dysplasia; IVH, intraventricular hemorrhage; PVL, periventricular leukomalatia; NEC, necrotizing enterocolitis; ROP, retinopathy of prematurity; LOS, late onset sepsis; wk, weeks of gestation.
Significant changes over time are set in bold.
See text for details.
